# The Mediating Roles of Emotional Regulation on Negative Emotion and Internet Addiction Among Chinese Adolescents From a Development Perspective

**DOI:** 10.3389/fpsyt.2021.608317

**Published:** 2021-04-09

**Authors:** Lijuan Liang, Mingrui Zhu, Jiali Dai, Min Li, Ya Zheng

**Affiliations:** ^1^Department of Psychology, The First Affiliated Hospital, Hainan Medical University, Haikou, China; ^2^Department of Neurology, The People's Hospital of Liaoning Province, Shenyang, China; ^3^Daqing No.3 Hospital, Daqing, China; ^4^HeBei Institute of International Business and Economics, Qinghuangdao, China; ^5^Department of Psychology, Dalian Medical University, Dalian, China

**Keywords:** depression, anxiety, stress, internet addiction, expressive suppression, cognitive reappraisal

## Abstract

Previous researches indicated that emotional regulation can be associated with depression and anxiety, which may be an important mediating factor between emotional regulation and internet addiction. However, the mechanism between these associations has received little attention and it is still unclear. This study has examined 716 Chinese adolescents, 341 were males (47.6%), aged 13 to 18(Mean = 14.58, SD = 1.50), using a cross-sectional survey involving Young's Diagnostic Questionnaire for Internet Addiction, the nine-item Patient Health Questionnaire (PHQ-9), the seven-item Generalized Anxiety (GAD-7) scale, and the Emotion Regulation Questionnaire (ERQ). Correlation analysis, multiple-group analysis and structural equation modeling were carried out in SPSS Statistics version 23 (IBM, Armonk, NY) and AMOS version 21. Cognitive reappraisal had a significantly negative direct effect on Internet addiction (β = −0.118, *p* < 0.05). Furthermore, negative emotions mediated the relationships between expression suppression and Internet addiction [β = 0.149, 95% CI = (0.099, 0.212)] and the relationship between cognitive reappraisal and Internet addiction [β = −0.101, 95% CI = (−0.147, −0.065)]. The differences in the structure path coefficients for different development stages demonstrated that recognitive reappraisal showed more protective roles for negative emotion (*p* < 0.01), and negative emotion also predict Internet addiction more effectively in high school students (*p* < 0.001). However, cognitive reappraisal directly predicted negative Internet addiction in junior high school students. Therefore, the intervention on adolescents for internet addiction should not only focus on emotional regulation and negative emotion, but also development stages of adolescents.

## Introduction

With the development of society and information technology, the internet has brought convenience, but also some problems, especially for adolescents. Physical and psychological characteristics show that adolescents have relatively poor self-control and immature psychology and behavior. Adolescents may be prone to internet addiction (IA), which can affect their physical (1) and mental health (2) and academic performance (3), even generating suicidal ideation (4). Internet addiction is characterized by psychological dependence, tolerance, and withdrawal symptoms and is included in the “Internet Addiction” chapter of the Diagnostic and Statistical Manual of Mental Disorders, Fifth Edition (DSM-V; American Psychiatric Association, 2013) (5). The prevalence of internet addiction among Chinese adolescents was found to be 10.4% (6). Many adolescents with internet addiction show more problematic internet usage, which may be associated with significantly more negative emotion and greater deficits in emotional regulation (7). There are obvious differences between boys and girls in Internet addiction. For example, adolescents with masculine temperament prefer competitive games, while adolescents with feminine temperament show low preference for competitive games. However, due to the virtuality of cyberspace, there may be cross gender behavior in adolescents' online behavior (8). Meanwhile, adolescents experience more negative emotions, which may be related to the contradiction between the demand for emotional autonomy and immature emotion regulation in adolescence (9). However, fewer studies simultaneously focused on the mediating roles of cognitive reappraisal and expressive suppression in internet addiction and the model invariance across gender and age.

### Depression, Anxiety and Internet Addiction

Owing to factors that pertain to academic performance (10), interpersonal problems (11), and family (12), many adolescents suffered from emotion problems, especially negative emotion of depression and anxiety (13). Many studies have considered that depression and anxiety may be the main risk factors for internet addiction among adolescents (14, 15). Internet addiction should be a negative coping style of avoiding problems. Therefore, the behavior of internet addiction may further aggravate the symptoms of negative emotions. The internal mechanism between emotion problems and internet addiction has played an important role in intervention and the treatment of internet addiction (16, 17). A possible explanation for these associations is that individuals with depression and anxiety may try to self-regulate emotional states through internet addictive behaviors (18). Positive emotional regulation strategies may be mediating roles between emotion problems and internet addiction.

According to Cognitive Behavior Theory (CBT), anxiety and depression of adolescents may be the mediator variables between emotional regulation and problem behavior (19). The problem behaviors of adolescents mainly include internalization and externalization. Internalization problems include all kinds of over-inhibited or inward-directed behavior, such as anxiety and depression, while externalization problems include all kinds of uninhibited or outward-focused behavior, such as internet addiction and aggression. Therefore, internet addiction as an externalized behavior may also be influenced by emotion regulation, but few researches have explored the relationship and psychological mechanism between emotion regulation and internet addiction.

### The Mediating Role of Expressive Suppression and Cognitive Reappraisal

Emotional regulation deficits have been theoretically and empirically associated with affective disorders and addiction. Some studies have tried to intervene in internet addiction in adolescents with cognitive behavioral therapy (CBT) (20). Cognitive factors may be key factors in internet addiction. Some studies have supported the notion that the ability to regulate emotions may predict internet addiction, and therefore producing changes in cognition may be an effective intervention method (21, 22). Cognitive reappraisal, as an adaptive strategy, is defined as cognitively transforming a situation in order to modify its impact on one's emotions (23). Cognitive reappraisal may help individuals to re-evaluate a situation of maladjustment and improve the ability of self-control. However, expressive suppression is a maladaptive strategy defined as inhibiting emotion expressive behavior (24). Some studies have shown that adolescents with internet addiction had greater difficulty in emotional regulation, manifested by excessive expressive suppression and too little cognitive reappraisal (25, 26). Owing to excessive suppression of negative emotional experiences, the correlation between negative and internet addiction may be enhance. Therefore, a deficit of emotional regulation may further strengthen the association between negative emotions and internet addiction.

According to previous studies, depression (27) and anxiety (28) are positively associated with internet addiction, and emotional regulation may have a mediating role between negative emotion and internet addiction.

### The Effect of Age and Gender

Many related studies have shown that internet addiction and emotional features of adolescents vary with age and sex (29). Although the related research supports the fact that negative emotion, such as depression and anxiety, play important roles in the severity of internet addiction, gender also plays an important role in the structural equation model (30). In addition, compared with boys, girls were found to be at a higher risk of mood symptoms only and of comorbid IA and mood symptoms (13). However, few studies have referred to the invariance of the structural model relating negative emotion and internet addiction, and some research shows that the path relating emotion and internet addiction shows no difference, which assumed negative emotion as mediator between school climate problematic internet use and ignore the comparison of progressive equivalence models (31). According to the different psychological mechanisms of age and gender, the treatment of internet addiction should consider the variables of age (32) and gender (33, 34).

### The Present Study

Emotion problems, such as depression (35) and anxiety (36) have been associated with internet addiction, so emotion problems may be risk factors for internet addiction. Expressive suppression may be a risk mediating factor between emotion problems and internet addiction. Internet addiction is affected directly by depression and anxiety, and indirectly by expressive suppression and cognitive reappraisal. Given the mediating effects of gender and age, we also considered the roles of gender and age in this model to determine whether the model developed for all participants was suitable for all adolescents regardless of age and gender.

## Materials and Methods

### Participants

A total of 716 adolescents participated in the study. The valid data set comprised 690 participants after deleting invalid data, and the data efficiency was 96.37%. All adolescents gave informed consent before starting to fill out the form by choosing whether agree to participate in this study or not.

### Procedure

The participants in this study were students who completed a self-reported questionnaire from the provinces of Sichuan and Hainan. They were first asked to read an informed consent declaration and entered the survey only if they agreed by QR (quick response) code or web page. They could opt to remain anonymous or use their real name when filling in the questionnaire.

### Ethics Statement

Ethical approval for this study was obtained from the Hainan Medical University Ethics Committee (HYLL2020005). Parents and schools were informed to obtain the consent prior to the study. All participants were volunteered to participate in the study and receive individual psychometric results at the end of the measurement.

### Measurement

#### Depression

The nine-item Patient Health Questionnaire (PHQ-9) has been widely utilized to assess symptoms of depression (37). The nine items of the PHQ correspond to the diagnostic criteria of depressive symptoms in DSM-V. The PHQ-9requiresparticipants to self-report the frequency of related symptoms over the past 2 weeks. As a severity measure, the PHQ-9 score can range from 0 to 27, because each of the nine items is scored from 0 (not at all) to 3 (nearly every day). Cronbach's alpha for the PHQ-9 was 0.911 in the current sample.

#### Anxiety

The seven-item Generalized Anxiety Disorder (GAD) scale is generally utilized to measure anxiety symptoms among adolescents (38). The GAD asks participants to self-report on the status and frequency of symptoms during the last 2 weeks (39). The score for the GAD can range from 0 to 21, and the Cronbach's alpha was 0.924 among all participants in this study.

#### Expressive Suppression and Cognitive Reappraisal

The Chinese version of the Emotion Regulation Questionnaire (ERQ) consists of 10 items that measure two factors: expressive suppression (four items) and cognitive reappraisal (six items). Each item of the ERQ is scored one (completely disagree) to seven (completely agree). Scores of for all four expressive suppression items were summed to generate a single expressive suppression score, while scores for all six cognitive reappraisal items were added up to yield a single cognitive reappraisal scores (40, 41). The Chinese version of the ERQ shows good internal consistency, adequate validity in Chinese individuals. In the present study, the Cronbach's alpha coefficients for the expressive suppression and the cognitive reappraisal were 0.752 and 0.857, respectively.

#### Internet Addiction

Young's Diagnostic Questionnaire for Internet Addiction (YDQ) was applied to assess Internet addiction (42). The YDQ was modified according to the DSM-IV criteria for pathological gambling and consists of eight “yes” or “no” questions. The total score of the eight items ranged from 0 to 8, which also showed good reliability and validity in Chinese adolescents. Cronbach's alpha was 0.771 in this study.

### Data Analysis

First, we conducted descriptive statistics, Student's *t*-test, correlation analyses using SPSS Statistics version 23 (IBM, Armonk, NY). Next, Amos 21.0 was adopted to examine the hypothesized models. Structural equation modeling (SEM) was performed to test the mediating role of negative emotions in the relationships among expressive suppression, cognitive reappraisal and Internet addiction. Specially, The SEM analysis was conducted in two steps. Firstly, we tested the measurement model to examine whether the observed variables were properly chosen for the indicators of the latent variables. Secondly, we tested the structural model to examine the proposed associations among the latent variables. Indirect effects were also calculated using bias-corrected bootstrapping (5,000 bootstrap samples) with 95% confidence intervals (CIs) (43). The 95% CIs not including zero shows a significant effect. Furthermore, to assess the structural equivalence across gender and developmental stages, two multi-group (by adolescent gender or developmental stages of adolescents) SEMs were performed.

In the present study, several goodness-of-fit indices were adopted to assess the model-data fit. The first one was the Chi-square statistic and its associated *p* value. If the *p* value is not significant, it may show good model-data fit. However, the Chi-square statistic is sensitive to sample size (44). Therefore, we adopted the Chi-square to degrees of freedom ratio (χ^2^/df) to assess the model fit. A χ^2^/df ratio of <3 indicated a good model fit. Other substitutive indices were also used in the current study, including the Tucker-Lewis Index (TLI) (45), the comparative fit index (CFI) (46), the standardized root mean square residual (SRMR) (47), and the root mean square error of approximation (RMSEA) (48). A TLI and CFI larger than 0.95 and a SRMR and RMSEA <0.08 show good model fit (48). For the comparison of the nested models, differences in the χ^2^ (Δχ^2^) and the degree of freedom (Δdf) were employed to compare the models with the goodness of fit to test the model that best fit the data (49, 50). Specifically, the standard of comparison between the two nested models is as follows: when the degrees of freedom increase with a significant increase in the corresponding Chi-square value (that is, Δχ^2^/Δdf is significant), the better model is the one with a smaller degrees of freedom. Otherwise, the larger degrees of freedom model are better. The predictive and explanatory powers of the model were assessed using path coefficients and R^2^.

## Results

### Descriptive Statistics and *t*-Tests

The characteristics of the participants and the distribution of Interent addiction are shown in Table 1. The average age of the adolescents in the current study was 14.59 (SD = 1.50) years, and the age range was 12 to 18 years; the study included 332 boys (48.1%) and 358 girls (51.9%). According to the standard definition of Interent addiction, the prevalence of Interent addiction was 10.72% (*n* = 74) among the adolescent internet users in this study. There were no gender differences in expressive suppression, cognitive reappraisal, Internet addiction, while depression and anxiety yielded significant gender differences. Compared with boys, girls had higher scores on the depression and anxiety scales. Forthermore, there were no developmental stages differences in expressive suppression, cognitive reappraisal, and Internet addiction, while depression and anxiety yielded significant developmental stages differences. High school students showed higher level of depression and anxiety than the junior high school students (Table 1).

**Table 1:** Descriptive statistics among the variables.

**Variables**	**Gender**					**Developmental stage of adolescents**				
	**Girls (*****N*** **=** **358)**		**Boys (*****N*** **=** **332)**		***t***	**Junior high school students (*****N*** **=** **504)**		**High school students (*****N*** **=** **186)**		***t***
	**Mean**	**SD**	**Mean**	**SD**		**Mean**	**SD**	**Mean**	**SD**	
Expressive suppression	15.20	4.77	14.97	4.73	0.65	14.99	4.94	15.35	4.20	−0.95
Cognitive reappraisal	29.08	5.92	28.71	6.74	0.77	29.00	6.47	28.63	5.93	0.69
Depression	6.36	6.13	4.69	5.16	3.87^**^	5.09	5.57	6.83	6.02	−3.58^**^
Anxiety	6.19	5.11	4.92	4.56	3.43^**^	5.29	4.80	6.34	5.06	−2.51^*^
Internet addiction	1.65	2.38	1.72	2.71	−0.40	1.65	2.58	1.77	2.45	−0.54

* p < 0.05;

** *p < 0.01*.

### Correlation Analysis

For all participants, expressive suppression was significantly positively related to depression, anxiety and Interent addiction (*r* = 0.298, *p* < 0.001, *r* = 0.309, *p* < 0.001, *r* = 0.08, *p* < 0.05, respectively), while cognitive reappraisal was significantly negatively associated with depression, anxiety and Internet addiction (*r* = −0.243, *p* < 0.001 and *r* = −0.219, *p* < 0.001, *r* = −0.180, *p* < 0.001, respectively). In addition, depression and anxiety were significantly correlated to Internet addiction (*r* = 0.332, *p* < 0.001 and *r* = 0.323, *p* < 0.001, respectively) (Table 2).

**Table 2:** Correlation among the main variables for all sample.

	**Range**	**Mean + SD**	**1**	**2**	**3**	**4**	**5**
1. Expressive suppression	4–28	15.09 ± 4.75	–				
2. Cognitive reappraisal	6–42	28.91 ± 6.32	−0.003	–			
3. Depression	0–27	5.56 ± 5.74	0.298^***^	−0.243^***^	–		
4. Anxiety	0–21	5.58 ± 4.89	0.309^***^	–.219^***^	0.804^***^	–	
Internet addiction	0–8	1.85 ± 2.01	0.080^*^	−0.180^***^	0.332^***^	0.323^***^	–

* p < 0.05;

*** *p < 0.001*.

### Mediational Model Analysis

**Figure 1:**
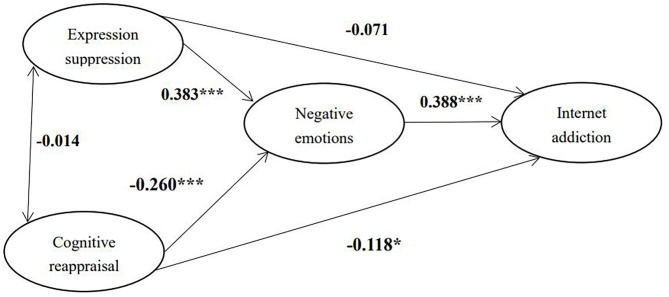
Standardized estimates of the mediated model. *p < 0.05; ***p < 0.001.

Firstly, the hypothesized measurement model contained 20 observed variables and five latent variables: expression suppression, cognitive reappraisal, depression, anxiety and Internet addiction (Figure 1). Depression and anxiety were loaded on the latent variable of negative emotion, while other all items were loaded on their corresponding latent variables in the measurement model. For example, the total eight items in the YDQ were loaded on the latent variable of Internet addiction. The measurement model was a good fit to the data, (χ^2^ = 275.551, χ^2^/df = 1.680, TLI = 0.969, CFI = 0.974, SRMR = 0.038, RMSEA = 0.032). The indicators loaded well-onto each latent variable with standardized factor loadings ranging from 0.470 to 0.903. When evaluating the structural model, we analyzed the significance of the entire model as well as the significance of the relationship and variances among the multiple factors in the model. According to the fit standards, our model fits well with the empirical data (χ^2^ = 275.551, χ^2^/df = 1.680, TLI = 0.969, CFI = 0.974, SRMR = 0.038, RMSEA = 0.032). Expression suppression had no significant direct effect on Internet addiction (β = −0.071, *p* > 0.05), while cognitive reappraisal had a significantly negative direct effect on Internet addiction (β = −0.118, *p* < 0.05). Furthermore, negative emotions mediated the relationships between expression suppression and Internet addiction [β = 0.149, 95% CI = (0.099, 0.212)]. In addition, negative emotions also mediated the associations between cognitive reappraisal and Internet addiction [β = −0.101, 95% CI = (−0.147, −0.065)]. Finally, we found that 17.2% variance of Internet addiction could be explained by this model.

### Structural Invariance of the Mediated Model Analysis

To assess the structural invariance of the mediated model across gender and developmental stages of adolescents, two nested models were estimated, respectively. The first model allowed the structure coefficient of the two models to be estimated freely according to gender, while the second model was conducted for the structure path coefficients to be equal. The results found that these two models were not significantly different, Δχ^2^[(5), *N* = 676] = 7.012, *p* = 0.220, indicating that they were not differed according to gender. In addition, the first model allowed the structure coefficient of the two models to be estimated freely according to the developmental stages of adolescents, while the second model was conducted for the structure path coefficients to be equal. The results found that these two models were also not significantly different, Δχ^2^[(5), *N* = 676] = 18.058, *p* = 0.003, indicating that they differed according to the developmental stages of adolescents. In addition, we utilized critical ratios of differences (CRDs) as an index to examine the differences in the structure path coefficients between junior high school students and high school students. If the CRD was larger than 1.96, then the associations between these two variables would demonstrate a significant developmental stages difference as *p* < 0.05. First, the results showed that the structure path from cognitive reappraisal to negative emotions revealed a significant developmental stages difference (CRD = −2.065, *P* < 0.05). More specifically, first, the path coefficient for junior high school students was β = −0.23, *p* < 0.01, while the path coefficient for high school students was β = −0.35, *p* < 0.01. Therefore, cognitive reappraisal had a far greater protective role against the negative emotions among high school students than junior high school students. Second, the results showed that the structure path from negative emotions to Internet addiction revealed a significant developmental stages difference (CRD = 3.057, *P* < 0.001). More specifically, the path coefficient for junior high school students was β = 0.28, *p* < 0.01, while the path coefficient for high school students was β = 0.70, *p* < 0.001. Therefore, negative emotions had a far greater prediction to Internet addiction among high school students than junior high school students. Finally, the results showed that the structure path from cognitive reappraisal to Internet addiction revealed a significant developmental stages difference (CRD = 2.711, *p* < 0.01). More specifically, the path coefficient for junior high school students was β = −0.19, *p* < 0.01, while the path coefficient for high school students was β = 0.11, *p* > 0.05. Therefore, cognitive reappraisal had a far greater direct prediction to Internet addiction among junior high school students than high school students. Thus, there were the structural invariance of the mediated model across gender, while the developmental stages of adolescents played the moderating effects in the structural paths in the mediated model.

## Discussion

### Gender and Age Differences

Our study showed that symptoms of depression and anxiety were significantly more common in girls than in boys. This result is consistent with most previous studies that female sex may be a risk factor for depression and anxiety (51). Among adolescents, the high school students showed significantly higher levels of depression and anxiety symptoms than junior school students, which may be related to more academic pressure, as well as emotional problems linked with physical and mental development.

### Correlation Among Main Variables

Expressive suppression showed significant positive correlation with depression, anxiety and Internet addiction. Depression and anxiety are highly comorbid, which is broadly characterized by an overreliance on expressive suppression. Previous researched also supported that expressive suppression was associated with negative emotion consequences (52). Negative emotion of depression and anxiety is also characterized by ineffective utilization of cognitive reappraisal, which inhibits the potential positive emotion. Similar with previous researches, cognitive reappraisal showed negative correlation to depression and anxiety (53, 54). The high school students may suffer from stressful or uncontrollable situations, due to underutilization of cognitive reappraisal. For negative emotion, treatment intervention may appear to increase cognitive reappraisal but decrease expressive suppression. In addition, expressive suppression was negative correlated with Internet addiction, but cognitive reappraisal was positively correlated with Internet addiction. The results were consistent with related research that emotion dysregulation may be a potential risk factor for Internet addiction (55). Because of the outbreak of COVID-19, adolescence may also have troubles, for example, lack of social communication, lack of peer communication and academic pressure. However, there are some difficulties in emotion regulation among some adolescents, which may enhance the negative emotion. Negative emotions may lead to externalized behaviors, such as Internet addiction. Therefore, dysregulated negative emotions in adolescents may be a risk factor for psychopathology, but appropriate emotion regulation strategy may be a protective factor for psychopathology (56).

### Mediating Effect of Negative Emotion

This study demonstrated that expressive suppression couldn't predict Interent addiction directly, cognitive reappraisal could predict Internet addiction directly. Negative emotion of depression and anxiety conducted mediating variables between emotional regulation and Interent addiction. On the one hand, similar to previous research, negative emotion was the partial mediating factor between cognitive reappraisal and Interent addiction (57–59). Negative emotion are important mechanisms in the relationship between emotional regulation and Interent addiction. The higher the score of cognitive reappraisal corresponded to the lower the negative emotion and the less the Interent addiction. On the other hand, depression and anxiety were also completely mediating factor between expressive suppression and Interent addiction. The higher the score of expressive suppression predicted the higher the negative emotion and the more the Interent addiction. Cognitive reappraisal allows individuals to reframe negative emotional situations and reassess emotional events, which enables them to have better adaptability to negative situations, and to gain more control (23). Expressive suppression is a negative coping style, which may enhance negative emotional experience, leading to deterioration of self-control, cognitive bias, and maladaptive behavior problems (24). At the same time, expressive suppression may also be harmful for an individual's social functioning and reduce the acquisition of social support (60, 61). For younger adolescents, there are a lot of social support from parents, teachers, friends and so on. Compared with older adolescents, they also confront with less academy stress. This study demonstrated that expressive suppression may be not the important mediating role between negative emotions and internet. Adolescents may still be more inclined to express their emotions rather than over restrain them. Cognitive reappraisal could be beneficial in enabling individuals to distinguish negative emotions, experience lower emotional intensity, and adopt more and various emotional regulation strategies. Research has shown that cognitive reappraisal reduced not only emotional experience but also bilateral amygdala activation (62).

### Invariance of the Model

To determine whether both different genders and different development stages of adolescents are applicable in the models, an invariance study of the structural models was conducted. The results showed that structural invariance across gender was accepted, but the invariance across different development stages of adolescents was not established. The structure models could be suitable for both boys and girls. Previous studies revealed that over-suppression of negative emotion may be harmful to the relief of negative emotions and lead to more behavioral problems and physical diseases, especially in females (63, 64). In our study, almost of adolescents are in the early stage of adolescents, psychological and behavioral differences between boys and girls haven't appeared particularly significance. With the development of adolescents, the differences may be more and more significant. As a result of social expectations, males are less likely to express themselves when they are faced with depression and anxiety, but are more willing to take on negative emotions and deal with bad situations by themselves.

According to invariance across different development stage of adolescents, the structure models couldn't also suitable for different stages of adolescents. Compared with junior high school students, cognitive reappraisal could be more protective factors for negative emotion of depression and anxiety among high school students. With the development of adolescents, cognitive function gradually improved, high school students could be better at cognitive reappraisal than junior high school students (65). The cognitive-affective process suggested that the improvement of cognitive level may be beneficial to the improvement of negative emotional experience. Similar with previous studies, anxiety and depression positively predicted Internet addiction of adolescents (15). However, negative emotion of high school students could be more effectively predicting roles in Internet addiction than junior high school students. Compared with junior high school students, high school students have more academic pressure, which is associated with more negative emotions. Finally, cognitive reappraisal is more effective in predicting Internet addiction of junior high school students. Related studies have demonstrated that emotion dysregulation is risk factors for Internet addiction. More cognitive reappraisal predicted greater reduction in negative emotion (66).

### Conclusion

According to cognitive behavior theory, emotional regulation plays important roles in Internet addiction. The way of emotion regulation affects emotional experience, which directly positively predicts Internet addiction. In this study, expressive suppression was a risk factor of negative emotion and Internet addiction, while expressive reappraisal was a protective factor of negative emotion and Internet addiction. Expressive suppression affects Internet addiction through negative emotions, while cognitive reappraisal affects Internet addiction directly. At the same time, there are some differences in the path coefficient of the model among the different development stages of high school students. This found reminds that psychological intervention should consider emotional regulation and negative emotion, but also development stages of adolescents.

## Limitations and Implications

Longitudinal study may be more beneficial to explore the psychological development mechanism between negative emotions and Interent addiction. Insufficient of older adolescents may affect this invariance of age, we should increase the sample of older adolescents in the future. According to the result, relief of depression and anxiety could be main intervention strategies to decrease Interent addiction for adolescent. In addition, cognitive reappraisal should be benefit for reducing of Interent addiction, which played a positive mediating role between depression, anxiety and Interent addiction.

## Data Availability Statement

The raw data supporting the conclusions of this article will be made available by the authors, without undue reservation.

## Ethics Statement

The studies involving human participants were reviewed and approved by Hainan Medical University. Written informed consent to participate in this study was provided by the participants' legal guardian/next of kin.

## Author Contributions

LL carried out experiments, conducted the statistical analysis, and wrote the manuscript. MZ, JD, and ML conducted data collection work and revised the manuscript. YZ designed experiments and critically reviewed the manuscript. All authors contributed to the article and approved the submitted version.

## Conflict of Interest

The authors declare that the research was conducted in the absence of any commercial or financial relationships that could be construed as a potential conflict of interest.
